# A Case of Obstructed Bowel

**Published:** 1886-09-20

**Authors:** G. G. Roy

**Affiliations:** Professor Materia Medica in Southern Medical College


### Southern Medical Record.

A MONTHLY JOURNAL OF PRACTICAL MEDICINE.

☞All Communications must be addressed to R. C. WORD, M. D., Managing Editor.

"*Quicquid Præcipies Esto Brevis.*"

VOL. XVI. ATLANTA, GA., SEPTEMBER 20, 1886. NO. 9

### ORIGINAL AND SELECTED ARTICLES.

### A CASE OF OBSTRUCTED BOWEL.

BY G. G. ROY, M. D.,
Professor Materia Medica in Southern Medical College.

I was called to visit Mrs. M., age fifty-nine, the night of the 3rd of May. Her previous health had been good, with the exception of occasional colic. I found her in the following condition: Temperature and pulse about 100; she complained of fullness of the abdomen with some pain and inability to get a free movement of the bowels. She casually remarked "that she had a lump in the groin, but she had several times before had the same lump, which disappeared of itself and gave her no trouble and but little concern."

The tension of the abdomen, the condition of the bowels, in connection with the "lump," aroused my suspicion and anxiety and induced me to give the "lump" a very thorough and careful examination.

I found in the inguinal region of the right side, below Poupart's ligament, a tumor, about the size of a hen's egg, movable, and having the firmness and other characteristics of one of the inguinal glands in an enlarged condition. It did not have the characteristic touch of a hernia or any other marked physical sign, but still my apprehensions were strong that it was.

For the purpose of relieving pain, and inducing rest, I gave an anodyne and applied hot cloths over the abdomen, and there being strong indications for a mercurial, I ordered, after several hours of rest—

℞. Ext. rhei.................................grs. ij,
Bluemass pill.................................grs. ij,
Pulv. ipecac.................................grs. ij,
Ext. hyoscy.................................grs. ij.

To be divided into six pills, one to be given every two hours.

The next morning I found that she had vomited frequently during the night; tongue dry, heavily coated with brownish fur; great thirst; no feaver or pain; no change in the tumor, and no action of the bowels.

A warm enema of soap and water with 2 ounces of castor oil and 1 drachm of spirits of turpentine was administered and soon evacuated, bringing only a small amount of green fecal matter. In a short while the warm water enema was repeated and passed unchanged. No change in the size or character of the tumor. The following combination was then used:

℞. Hydrarg. chlo. mit.............................grs. xij,
Sodæ bicarb.............................grs. xij,
Sac. pepsin.............................grs. vi.

M. ft. caps. 6. S. One every two hours.

Vomiting continuing, these were either ejected or failed to make any cathartic impression.

Calling again in the afternoon, I repeated the enematar. Attaching a large gum catheter to the pipe of a Davidson syringe and introducing into the bowels its full length, I injected nearly a gallon of warm water with 2 ounces castor oil, 1 drachm spirits turpentine and 5 drops of croton oil. This was evacuated unchanged, and in one hour repeated with the same result.

It is well to note that, after the first night, there was little or no fever, and but slight constitutional disturbance of any kind. At night calomel, soda, pepsin, and half a drop of croton oil was directed to be given every three hours when not asleep.

There was no appreciable change in the condition of the patient next morning, save more exhaustion from vomiting and want of rest. However, I imagined there was some softening and diminution in the size of the "lump" in the groin.

The enemata were renewed, and given in larger quantity and at shorter intervals. Freshly prepared cit. of magnesia was allowed *ad libitum* in the place of water. There being no appreciable improvement in the condition of the patient at noon, I called at the office of my friend, Dr. K. C. Divine, and give him the history of the case. Becoming interested, he very kindly offered to assist me in my dilemma. After a very careful examination, his conclusion as to the character of the tumor (like mine) was one of doubt. He thought it possibly an enlarged gland, its physical characteristics rather supporting this view, than that of hernia; but the inability to secure an evacuation after such persistent effort, with other symptoms (though not prcminent) indicated that it was a gut obstruction.

In our dilemma of doubt, we decided to renew the enemata, using more water with a long rectal tube and a change of position of the body of the patient. We first placed her on her left side with hips elevated and head lowered, and by means of the long rectal tube, introduced into the bowel almost its full length, over a gallon of hot soap-water was injected. This was retained fifteen or twenty minutes and then discharged unchanged, but we discovered there was considerable diminution in the size of the tumor. Thus encouraged, her position was again changed. Her hips and legs were elevated to an angle of about 45° (thereby relaxing the abdominal muscles and causing the bowels to gravitate downward toward the diaphragm) and placed upon an inclined plane, improvised by turning a chair "bottom upwards," nailing a strip across the ends of its "back legs," covering with a thick quilt and then placing the body and hips well upon the back of the chair with the legs suspended over the strip nailed across. In this position we injected over a gallon warm water, and on examination found that the "lump" in the groin had entirely disappeared. This enema was retained about half an hour and then discharged with some coloring of fecal matter.

Becoming much exhausted from this tedious manipulation, 20 drops F. E. cannabis indica were ordered; to be repeated in two hours if not asleep. The first dose gave her a good night's rest. We called at 7 o'clock, a. m., and found after a refreshing night's sleep, her respiration, circulation and temperature normal, but there had been no evacuation of the bowels. We ordered calomel, ʒ i; divide into three powders; one to be taken every two hours.

At my visit about noon, all the powders having been taken, and still no evacuation of the bowels, I ordered a palatable emulsion of ol. recini, ℥ iij; mucil. acaciæ, ad. ℥ vij, and a tablespoonful to be given every hour if borne by the stomach. Five doses were taken and at 5:30 o'clock a telephone message announced the "glad tidings," "that a large, green, fecal evacuation had passed, and what must now be done." I replied, "nothing," as Dr. D. and I expected to see her at 6 o'clock.

We found patient much relieved, comfortable and cheerful! abdomen much relieved and no unpleasant symptom present.

Patent has continued regularly to improve, having three or four good bilious actions during day and night without the use of medicine.

We feared troublesome hypercatharsis from the great quantity of purgative medicine given, but none occurred. All the functions having returned to their natural and healthy performance of duty, but being still quite feeble, she was discharged on the 12th with a tonic of wine of iron and wine of pepsin, and very strictly enjoined to keep her bowels in a laxative condition and to guard against and watch the slightest re-appearance of the tumor. I am now assured that this was an obstruction of the gut by a nuckle's being forced into the inguinal canal and impacted therein by a mass of hard fecal matter.

I would especially direct attention to the means and mode of relief, *i. e.*, the elevation of the hips to almost a right angle with the body, the distention of the bowels to utmost capacity by injections of hot water thrown as high up as the long rectal tube would reach. The water should be forced into the bowel very slowly and gradually.

Dr. D. informed me that he had occasion, just a few days previous to this, to resort to the same procedure in a case of inguinal obstruction of a negro man to whom he was called to perform herniotomy, and with the most gratifying success.

